# Effect of 1,3-Beta Glucans Dietary Addition on the Growth, Intestinal Histology, Blood Biochemical Parameters, Immune Response, and Immune Expression of CD3 and CD20 in Broiler Chickens

**DOI:** 10.3390/ani12223197

**Published:** 2022-11-18

**Authors:** Shimaa A. Amer, Ghadeer A. Attia, Abed Alsalam Aljahmany, Aya K. Mohamed, Amer Al Ali, Ahmed Gouda, Gehan N. Alagmy, Hend M. Megahed, Taisir Saber, Mahmoud Farahat

**Affiliations:** 1 Department of Nutrition & Clinical Nutrition, Faculty of Veterinary Medicine, Zagazig University, Zagazig 44511, Egypt; 2 Department of Medical Basic Sciences, College of Applied Medical Sciences, University of Bisha, 255, Al Nakhil, Bisha 67714, Saudi Arabia; 3 Department of Clinical Laboratory Sciences, College of Applied Medical Sciences, University of Bisha, 255, Al Nakhil, Bisha 67714, Saudi Arabia; 4 Animal Production Department, Agricultural & Biological Research Division, National Research Center, Dokki, Cairo 11865, Egypt; 5 Department of Pathology, Animal Health Research Institute (AHRI), Agriculture Research Center (ARC), Zagazig 44511, Egypt; 6 Department of Biochemistry, Animal Health Research Institute (AHRI), Agricultural Research Center ARC, Zagazig Branch, Zagazig 44511, Egypt; 7 Department of Clinical Laboratory Sciences, College of Applied Medical Sciences, Taif University, P.O. Box 11099, Taif 21944, Saudi Arabia

**Keywords:** broiler chicken, 1,3-β-glucans, antibiotic alternatives, growth, gut health, hormone profile, immunohistochemistry

## Abstract

**Simple Summary:**

Recently, finding alternative feed additives for antibiotics has become an increasing approach by animal nutritionists. This study evaluated the addition of 1,3-β-glucans as a potential growth promoter alternative to antibiotics in broiler chicken diets at the following four grading levels: 0, 50, 100, and 150 mg 1,3-β-glucans kg^−1^. The study concluded that 1,3-β-glucan could be included in broiler chicken diets for improving the development and integrity of the intestine and enhancing the bird’s immune status. Dietary 1,3-β-glucan has a hypolipidemic effect and improves the hormonal profile of birds without affecting their growth rate.

**Abstract:**

This experiment evaluated the impact of the dietary addition of 1,3-β-glucans (GLU) on broiler chickens’ growth, intestinal histology, blood biochemical parameters, and immunity. Two hundred three-day-old male broilers (Ross 308) (97.93 ± 0.19 g/chick) were randomly assigned into four treatments with five replicates, each containing ten birds, in a complete randomized design. The four treatments were formulated with 0, 50, 100, and 150 mg 1,3-β-glucans kg^−1^ in broiler chicken diets. During the study, no significant impacts (*p* > 0.05) were observed in weight gain and feed conversion ratio (FCR) between treatment groups. Based on the results of total body weight gain and FCR, the optimal level of 1,3-β-glucan is 120 mg Kg^−1^. The intestinal histomorphology was improved by GLU supplementation, as indicated by increased villi height and villi height to crypt depth ratio (*p* < 0.01). All levels of supplemental β-1,3 glucan decreased the serum total cholesterol (TC), triglyceride levels, and low-density lipoprotein cholesterol (LDL-C) (*p* < 0.05). The serum levels of growth hormones (GH), triiodothyronine (T3), and thyroxine (T4) were increased in GLU-supplemented groups (*p* < 0.05). The serum immune indices (lysozyme activity, interleukin 10 (IL10), complement 3 (C3), and total protein levels) were increased in the GLU-supplemented groups (*p* < 0.05). Dietary GLU up-regulated the immunoexpression of CD3 (T-cell marker) and CD20 (B-cell marker) in the spleen of birds (*p* < 0.01). It can be concluded that 1,3-β-glucan can be added to broiler chicken diets for improving the development and integrity of the intestine and enhancing the bird’s immune status. The optimal level for 1,3-β-glucan dietary supplementation was 120 mg Kg^−1^. Dietary 1,3-β-glucan has a hypolipidemic effect and improves the hormonal profile of birds without affecting their growth rate.

## 1. Introduction

“Antibiotic Free,” “No Antibiotic Ever,” and “Raise Without Antibiotic” are new patterns of production techniques in the poultry industry [1]. Antibiotics have been used as growth promoters for decades. Still, most of them have been banned because of residual contamination in animal production and the expansion of resistant pathogenic bacteria, which has severe implications for the future efficacy of these essential drugs [2,3]. Therefore, finding alternate strategies, mainly using high-condition animals, feeding practices, and nutrition, to sustain animal performance and protect animals from the disease [4,5].

Prebiotics are one of the possible feed additives that control the intestinal ecology, intestinal microbiota, and host immunity. The term “prebiotic” refers to an element that has been selectively fermented and alters the intestinal microbiota’s structure and activity, promoting the host’s health and well-being [6]. Prebiotics are non-digestible food components that enable saprophyte bacteria growth [7,8].

One of the most popular prebiotics, beta-glucan (GLU), has a variety of biological activities, including immune role enhancement, anti-infection, and glucose regulation [2]. β-glucans are the groups of dietary fiber polysaccharides joined by D-glucose monomer through glycosidic bonds. β-glucans have three distinct glycosidic linkages such as β-(1,3)/β-(1,4)/β-(1,6), which are extracted from a range of natural sources, including the cell walls of bacteria, fungus, yeast, algae, and oats [9]. The pharmaceutical and functional feed industries are also considering GLU as a biological response modulator due to its positive effects on human and animal health. Their biological effects are affected by molecular mass, the tertiary structure, and the degree of branching [10]. β-glucans have been demonstrated to enhance intestinal health in poultry exposed to a bacterial challenge [11], promote macrophage activity [12], increase antibody titers following vaccination [13], and have anti-inflammatory and immune-modulating effects [14]. However, branching degree and molecular weight can affect the immunological response of β-glucan by enhancing anti-bactericidal and phagocytic activity and producing cytokines [15]. Additionally, intestinal macrophages could detect β-glucans, producing pro-inflammatory cytokines such as IL1 and tumor necrosis factor-alpha and increasing their phagocytic capacity [16,17]. Previous studies showed that β-glucan induced specific IgA in the intestine of chickens to protect them from pathogen infections [11,18].

Supplementing diets with 1,3-β-glucan has boosted immune cell activation and migration to the gut in chickens [19]. Other studies have observed improvements in bacterial infection resistance [20,21], improved intestinal health following coccidiosis infection [14,19], and overall improvements in growth rates and feed conversion efficiency [19]. Moreover, supplementation with 1,3-β-glucan enhances the vaccination response in broiler chicks, possibly because it increases innate immune activity [22]. We hypothesized that 1,3-β-glucan could improve the bird’s growth by altering the intestinal morphology, hormonal profile, and immune status of birds. Therefore, this study aimed to assess the effects of the dietary addition of different levels of 1,3-β-glucan on the growth performance, hormonal profile, intestinal histology, immune status, and immune expression of CD3 and CD20 in broiler chickens.

## 2. Materials and Methods

### 2.1. Animal Ethics

The present study was performed in an animal experiment unit in the faculty of veterinary medicine at Zagazig University, Egypt. The experimental design and procedures used in this study were approved by the guidelines of the Committee of Animal Welfare and Research Ethics Zagazig University Egypt (Approval No. ZU-IACUC/2/F/153/2022).

### 2.2. Birds, Housing, Diets, and Experimental Design

One-day-old male broiler chicks (*n* = 200, Ross308 broiler) were purchased from a commercial chick producer. Birds were presented with a three-days adaptation period before the start of the experiment to reach an average body weight of 97.93 ± 0.19 g/chick. Then, chicks were randomly allocated to four groups with five replicates (10 birds/replicate) in a completely randomized design. Birds fed on four experimental diets, which consisted of basal diets supplemented with four levels of 1,3-Beta Glucans (AletaTM, KEMIN Industries, Scott Ave Des Moines, IA, USA); 0, 50, 100, and 150 mg GLU kg^−1^ diet (GLU0, GLU50, GLU100, and GLU150, respectively) for 35 days. All birds were allowed free access to feed and clean water during the experiment. Throughout the experiment, chicks were raised in the same management, sanitary and environmental conditions. The managerial conditions, vaccine program, and experimental diets were performed following ROSS 308 broiler nutrition specifications AVIAGEN [23]. The proximate composition of the experimental diets is shown in Table 1. The feed was offered in a mash form.

### 2.3. Growth Indices

On the 4th day of life, birds were individually weighed to record their initial body weight. Then the body weight (BW), body weight gain (BWG), and the amounts of feed intake (FI) were determined at the end of each feeding period; starter (0–10 days), grower (11–23 days), and finisher period (24–35 day). The feed conversion ratio (FCR) was calculated as follows: $\mathrm{FCR}=\frac{\mathrm{FI}\;\left(g\right)}{\mathrm{BWG}\;\left(g\right)}$.

### 2.4. Intestinal Histology

At the end experiment, samples from the small intestine (duodenum, jejunum, and ileum) (*n* = 3, 2 Cm) were taken from each group and preserved in 10% neutral buffered formaldehyde for 72 h, dehydrated, cleared, embedded in wax, sliced with a microtome (Leica RM 2155, Wetzlar, Germany) into 4 µm cross-sections and longitudinal sections, and stained using hematoxylin and eosin (H&E) [24]. The morphometric measures were determined as described by Amer, et al. [25].

### 2.5. Sampling

Ten birds/groups were chosen randomly and euthanized using cervical dislocation [26]. Blood was collected without anticoagulant to obtain serum samples, left to clot at room temperature, and centrifuged for 15 min at 3500 rpm. The serum was stored at −20 °C till biochemical analysis. Spleen samples were taken for immunohistochemistry. Small intestine samples were taken for histological assessment.

### 2.6. Serum Hormones Profile

Serum levels of Triiodothyronine (T3), Thyroxine (T4), growth, and leptin hormones were measured using chicken Elisa kits (Cat. No. MBS269454, MBS265796, MBS266317, and MBS025331, respectively, My Biosource Company in San Diego, CA, USA). The serum glucose levels were detected as described by Trinder [27].

### 2.7. Serum Lipid Profile and Protein Gram

Serum total cholesterol (TC), triglycerides (TG), and high-density lipoprotein cholesterol (HDL-C) were determined using Bio-spectrum colorimetric diagnostic kits (Egyptian Company for Biotechnology, Cairo, Egypt). Low-density lipoprotein-cholesterol (LDL-C) level was determined following the Iranian formula; LDL-C = TC/1.19 + TG/1.9 − HDL/1.1 − 38. Very low-density lipoprotein cholesterol (VLDL-C) was estimated using the turbidimetry method [28].

The serum total protein and albumin levels were estimated according to Grant [29] and Doumas, et al. [30], respectively. The serum level of globulin was determined mathematically by subtracting albumin values from total proteins [31].

### 2.8. Immune Indices

The serum lysozyme activity was assessed according to Lie, et al. [32]. The serum level of complement 3 was measured using a sandwich enzyme-linked immunosorbent assay (ELISA) kit (CAT. NO. LS-F9287Life Span Biosciences, Inc., Seattle, WA, USA)). The serum interleukin 10 (IL10) level was evaluated using chicken ELISA kits of MyBioSource Co., San Diego, CA, USA, CAT.NO. MBS701683.

### 2.9. Immunohistochemistry

At the end of the experiment, 3 samples of spleen per each group were collected to examine CD3 and CD20 immune expression in the spleen, according to Saber, et al. [33]. Slides were treated with mouse anti-Chicken CD3, clone CT-3 (Bio-Rad Lab., Dubai, United Arab Emirates), and CD20 (ThermoFisher Scientific, Waltham, MA, USA) and examined as described by Amer, et al. [25]. The average grayscale is used to express Immunoreactive intensity [34].

### 2.10. Statistical Analysis

Welch’s ANOVA was applied for variables that violated the homogeneity condition. While the ANOVA test was applied based on polynomial orthogonal contrasts. Linear and quadratic regression equations were calculated using SPSS Version 17 for Windows (SPSS Inc., Chicago, IL, USA). Pens were the experimental units for all analyses. Significant results followed Tukey’s honestly significant difference test. Variation in the data was expressed as pooled SEM, and the significance level was set at *p* < 0.05. The broken-line model with Tukey’s test considered data on total BWG and FCR for calculating the optimal supplementation level of dietary 1,3-β-glucan [35].

## 3. Results

### 3.1. Growth Performance

**Figure 1:**
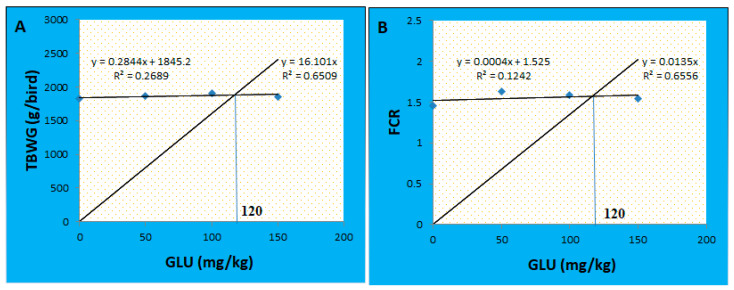
Broken line regression model showing the optimal level of 1,3-β-glucan supplementation based on the data of total body weight gain (**A**) and FCR (**B**).

During the starter period (4–10 days), no marked variations were detected between the groups concerning the BW, BWG, FI, and FCR (*p* > 0.05). Through the growth period (11–23 days), the FI was quadratically increased in the GLU100 group compared with the GLU0 group (*p* < 0.05). The GLU50 and GLU150 groups showed no significant (*p* > 0.05) differences in the feed intake compared with the GLU0 group. No significant changes were recorded in the BW, BWG, and FCR between the groups (*p* > 0.05). Through the finisher period (24–35 days) and the allover performance (4–35 days), no significant changes were recorded between the groups concerning the BW and BWG (*p* > 0.05). At the same time, the finisher feed intake and FCR were quadratically increased in the GLU50 group (*p* < 0.05). The total feed intake and FCR were quadratically increased in the GLU50 and GLU100 groups (*p* < 0.05) (Table 2). The final BW increased by 2.16, 4.40, and 1.65% for the GLU50, GLU100, and GLU150 groups, respectively. The BWG increased by 2.36, 4.70, and 1.80% for the GLU50, GLU100, and GLU150 groups, respectively. The feed intake increased by 14.14, 14.09, and 7.11% for the GLU50, GLU100, and GLU150 groups, respectively. Based on the total BWG and FCR, the optimal level of 1,3-β-glucan was 120 mg Kg^−1^ (Figure 1).

### 3.2. Intestinal Histomorphology

**Figure 2:**
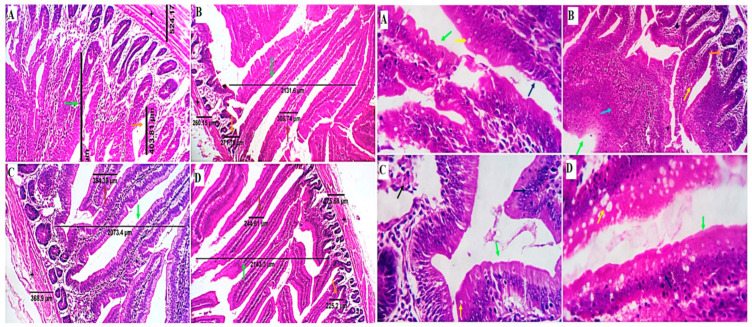
Photomicrographs from the small intestine (duodenum) of different experimental groups of chicken received 0 mg/kg (**A**), 50 mg/kg (**B**), 100 mg/kg (**C**), and 150 mg/kg (**D**) of GLU showing intestinal villi, crypts of Lieberkühn, submucosa, and muscle coat with normal histological structures. The dimensions of the villus length (green arrows), villus width (brown arrows), the crypt of Lieberkühn length (orange arrows), and muscular coat thickness (black stars). The estimated counting of goblet cells (yellow arrows) by routine H&E revealed increased goblet cell number in the GLU150 group and a decrease in the GLU100 group in comparison with the GLU0 and GLU50 groups. Mild villus epithelial stratification with multilayered proliferate cells can be observed in all groups, particularly group B (blue arrows). H&E × 100, 200.

The morphometric measures of the small intestine are displayed in Table 3 and Figure 2. The villus height (VH) in the duodenum was not significantly different between all treatments (*p* > 0.05). The jejunal VH was quadratically increased in the GLU50-100 groups (*p* < 0.01), while in the ileum, it was linearly increased in the GLU100 and GLU150 groups (*p* < 0.01) compared to the GLU0 group. The villus width (VW) in the duodenum and jejunum was decreased in all GLU-supplemented groups (*p* < 0.01) compared to the GLU0 group. The VW in the ileum was increased in the GLU100 group and reduced in the GLU150 group (*p* < 0.01) compared to the GLU0 group. The crypt depth (CD) and muscular coat thickness (M.C.T.) of the duodenum and ileum were decreased in all GLU-supplemented groups (*p* < 0.01) compared to the GLU0 group. The CD of the jejunum was decreased in all GLU-supplemented groups except for the GLU150 group, which was significantly increased (*p* < 0.01) compared to the GLU0 group. The M.C.T. of the jejunum was increased in the GLU50 and GLU150 groups and decreased in the GLU100 group compared to the control group. The VH:CD ratio was increased in the GLU100 group in the duodenum, the GLU50 and GLU100 groups in the jejunum, and the GLU100 and GLU150 groups in the ileum. The goblet cell count (GCC) in the duodenal sections revealed an increase in the GLU150 group and a decrease in the GLU100 groups in comparison with the GLU0 and GLU50 (*p* < 0.01). The GCC in the jejunum increased in GLU-supplemented groups (*p* < 0.01). The GCC in the ileum linearly increased in the GLU150 group and decreased in the GLU100 group compared to the GLU0 group (*p* < 0.01).

### 3.3. Serum Hormones Profile

The serum levels of T3 and T4 were linearly increased in the GLU-supplemented groups in a level-dependent manner (*p* < 0.01). Linear and quadratic increases in the serum growth hormone level were detected in the GLU-supplemented groups (*p* < 0.05). No significant differences were detected in the serum levels of glucose and leptin between all groups (*p* > 0.05) (Table 4).

### 3.4. Lipid Profile

The results showed a linear and quadratic decrease in the serum TG and LDL-C, a quadratic decrease in the serum TC, and a linear decrease in the serum HDL-C in the GLU-supplemented groups (*p* < 0.05). The serum VLDL-C levels were not significantly different between all groups (*p* > 0.05) (Table 5).

### 3.5. Immune Status

Linear and quadratic increases in the serum levels of lysozymes and IL10 and a linear increase in the C3 and total protein were detected in GLU-supplemented groups compared to the GLU0 group (*p* < 0.05). However, no significant changes were detected in the serum albumin and globulin levels between the groups (*p* > 0.05) (Table 6).

### 3.6. Immunohistochemistry

**Figure 3:**
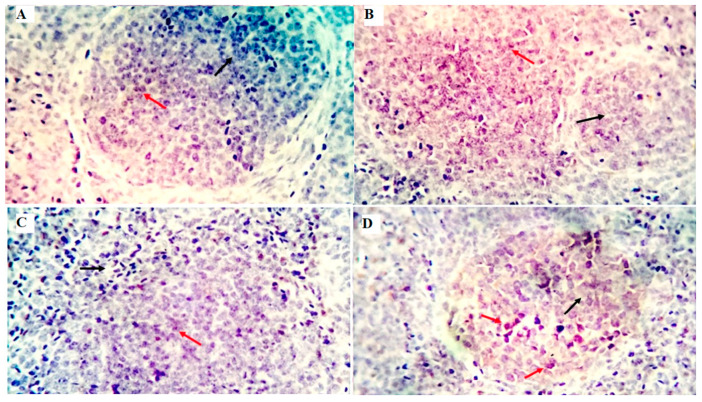
Shows the immunostained positive CD3 cells (red arrows) and negative cells (black arrows) in the spleen of different experimental chicken groups. The 0 mg/kg (**A**), 50 mg/kg (**B**), 100 mg/kg (**C**), and 150 mg/kg (**D**) of GLU.

**Figure 4:**
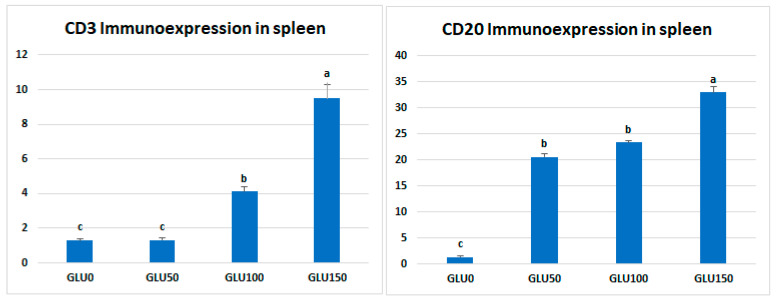
Morphometric analytic data of CD3 and CD20 (%) in the spleen of different experimental chicken groups. The 0 mg/kg (GLU0), 50 mg/kg (GLU50), 100 mg/kg (GLU100), and 150 mg/kg (GLU150). ^a,b,c^ Means carrying different superscripts are statistically different at *p* < 0.05.

**Figure 5:**
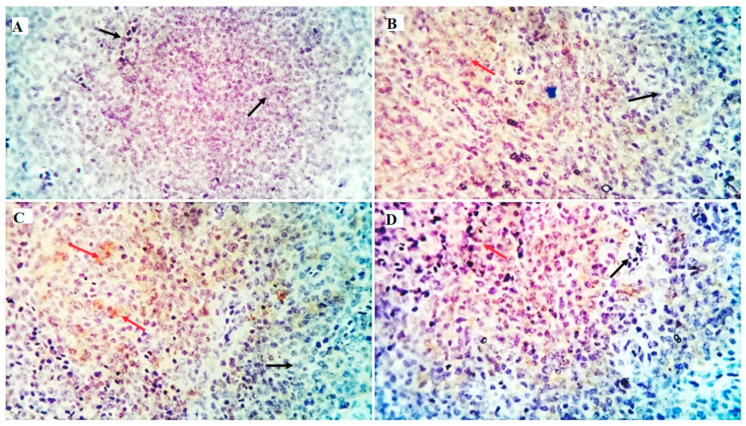
Shows the immunostained positive CD20 cells (red arrows) and negative cells (black arrows) in the spleen of different experimental chicken groups. The 0 mg/kg (**A**), 50 mg/kg (**B**), 100 mg/kg (**C**), and 150 mg/kg (**D**) of GLU.

The immune cell populations in the spleen of broiler chickens receiving 0 mg/kg (A), 50 mg/kg (B), 100 mg/kg (C), and 150 mg/kg (D) of GLU have been investigated using leukocyte-specific markers (CD3 and CD20). Average percentages of positive cells per 3 high power fields (HPF) to the CD3 T-cell marker were observed in different experimental groups as follows: 1.3, 1.6, 4.5, and 10.99% for the GLU0, GLU50, GLU100, and GLU150 groups, respectively (Figure 3 and Figure 4). Average percentages of positive cells per 3 HPF to the CD20 B-cell marker were observed in different experimental groups as follows: 1.5, 21.6, 24.1, and 35% for the GLU0, GLU50, GLU100, and GLU150 groups, respectively (Figure 4 and Figure 5).

## 4. Discussion

### 4.1. Growth Performance

The current study showed no changes in the BWG, FI, or FCR as a result of feeding on diets supplemented with GLU from 4 to 10 days. From 11 to 23 days, the BW, BWG, and FCR were not affected by the supplement, while the feed intake increased in the GLU100 group. Through the finisher period (24–35 days) and the allover performance (4–35 days), no significant changes were observed in the BW and BWG, while the finisher feed intake and FCR were increased in the GLU50 group. The total feed intake and FCR were increased in the GLU50 and GLU100 groups. This means birds fed on GLU-supplemented diets eat more but do not convert this feed to weight gain. This could be assumed due to the low energetic contribution of the β-glucans, even though it is thought that their contribution boosts birds’ innate immunity [36]. Another study proposed that decreased nutrient intake and reduced nutrient utilization efficiency were responsible for the reduced performance caused by β-glucan supplementation.

Moreover, the origin, structure, and molecular weight of the 1,3/1,6-glucan are three variables that can change the supplementation efficacy [37]. Furthermore, the nonsignificant effects of β-glucan on birds’ growth may result from energy rearrangement to immune enhancement, resulting in ineffective use of nutrients during growth. Other factors such as analysis of β-glucan, composition, dosage, purity, species, strains, and the occurrence of challenges also contribute to these different findings [21]. According to Rathgeber et al. [38], yeast β-glucan was equally as effective as the antibiotic virginiamycin, frequently added to broiler feeds at subtherapeutic doses, stimulating broiler chickens’ growth. Similarly, Zhang et al. [37] claimed that adding β-glucans can enhance the average daily gain and FCR. Other researches have shown that β-glucan improves the broiler’s performance [39,40]. Zhang et al. [37] informed that the optimal amount of β-glucan supplement is between 25 and 200 mg/kg to maximize the performance of broilers.

### 4.2. Intestinal Histomorphology

Evaluation of intestinal histomorphology is a crucial factor that may impact nutrient absorption and is considered a central defense barrier against pathogens [41]. Dietary characteristics such as feed particle size and composition may significantly impact intestinal morphology [42]. Decreased CD and increased VH can encourage nutrient digestion and absorption [43]. The crypt of the intestinal epithelium is a growing slot that promotes cell differentiation and maturation in the villi. A deeper crypt suggests rapid tissue turnover and possibly increases the need for new tissue [44]. The current study showed improved intestinal histomorphology of birds fed GLU-supplemented diets, as represented by long villi, a short CD, and an increased villus height to crypt depth ratio. The VH:CD ratio was raised in the GLU100 group in the duodenum, the GLU50 and GLU100 groups in the jejunum, and the GLU100 and GLU150 groups in the ileum. The goblet cell count was increased at the higher level of β-glucans (150 mg Kg^−1^). These results showed that β-glucan could enhance nutrient utilization and absorption by increasing the villi length and so the surface absorptive area of the intestine.

β-glucan improves the intestinal barrier mechanism and maintains the mucous membrane integrity by promoting the development of neurotransmitters involving close-linked protein, serotonin substance P, and acetylcholinesterase in the intestinal epithelium [45]. In addition, it improves the gastrointestinal tract’s ability through the development of intestinal peristalsis motility and promotes the diversity and richness of the intestinal microbiota using intestinal bacteria for fermentation [46]. Although dietary GLU improved most of the morphometric measures of the intestine that would enhance nutrient absorption and utilization, the growth performance was not influenced; this may be attributed to the energy contribution of GLU being rearranged for immune enhancement, resulting in ineffective use of nutrients during growth. Moreover, our results showed mild villus epithelial stratification with multilayered proliferating cells can be observed in all groups, particularly the GLU50 group. Such epithelial stratification could be a protective mechanism and or an overload regenerative process. It may increase the intestinal antimicrobial defensive mechanism [47]. Ding, et al. [48] reported increased VH, CD, and density of goblet cells in β-glucan-fed chicks, while no alterations were reported in the thickness of the muscle coat or intestinal wall. Morales-Lópezh et al. [49] showed an increased jejunal VH of chickens fed pure 1,3-1,6 β-glucan. Fasina and Olowo [44] showed morphometric differences in the jejunum and ileum but not in the duodenum by dietary supplementation with β-glucan. Shao et al. [11] showed that yeast–glucan-supplemented groups had higher jejunal VH and VH as follows: CD ratios than non-supplemented groups.

### 4.3. Lipid and Hormones Profile and Proteinogram

The obtained results revealed that all levels of supplemental GLU decreased the TC, LDL-C, and serum triglyceride levels, proving the hypolipidemic effect of supplemental GLU. Similarly, in broiler chickens, Elrayeh and Yildiz [50] reported reduced serum total cholesterol and triglyceride levels by dietary β-glucan supplementation. Chen [51] observed decreased serum cholesterol levels in broilers after adding chicory-based fructans to the diets. The hypolipidemic effect of dietary GLU was also observed in rabbits [52]. The hypolipidemic effect of GLU can be explained in three mechanisms; firstly, β-glucan can up-regulate the expression of p-AMPK and p-AKT and down-regulate gene expression of cholesterol synthesis, glycerolipid synthesis, fatty acid biosynthesis, and gluconeogenesis [53]; secondly, β-glucan combines with bile acid leading to the reduction of cholesterol levels in the body; thirdly, β-glucan can increases the gastrointestinal tract viscosity and delayed gastric evacuation along with carbohydrate absorption, so dropping the blood sugar and cholesterol levels [54].

The thyroid gland secretes T3 and T4, which are crucial for regulating several metabolic pathways and feeding metabolism. T3 and T4 hormones affect growth rate via various metabolic processes [55]. Elevated serum levels of thyroid hormones can be translated into better BWG [56]. Growth hormone (GH) is produced and released by the anterior pituitary gland’s somatotroph cells and is involved in several biological activities. GH regulates animal growth and development on a tissue level and throughout the body [57,58]. Other organ and tissue functions are controlled by GH when it binds to its receptor. Hepatic metabolism, which is regulated by GH, includes the lipid metabolism of adipose tissue and the liver [59]. The current study showed a significant increase in the serum levels of T3, T4, and GH in the GLU-fed birds in a dose-dependent manner. Despite the increased levels of T3, T4, and GH in the GLU-fed birds, the growth rate of these birds was not affected by the GLU dietary addition. This may be explained by the energy produced by GLU feeding may be transferred for immune activity and intestinal development instead of increasing the birds’ weight [60]. In addition, dietary GLU increased the birds’ feed intake with no change in the FCR compared with the unsupplemented group, which confirms the previous explanation. Chicken leptin significantly reduces feed intake and insulin secretion and thus may have a crucial role in regulating nutrient utilization [61].

In this study, serum glucose and leptin levels did not differ between groups. Besides, the serum total protein level was increased in the GLU-supplemented groups. Other results showed an insignificant effect of β-glucans on total proteins, albumin, globulin, and reduced glucose concentration in the blood serum of growing rabbits [52]. Tao, et al. [62] and El-Sawy, et al. [63] noticed no effect of yeast β-glucan supplementation on blood levels of total proteins or albumin.

### 4.4. Immune Status

The body fights off the pathogen invasion through specific and non-specific immune mechanisms. Cytokines are essential to balancing immunological and inflammatory responses, which control innate and adaptive immune responses to antigens and infectious pathogens. Anti-inflammatory cytokines defend against intestinal inflammation, while pro-inflammatory cytokines modulate the host’s inflammatory response to reduce vulnerability to infection [64]. Antigen presentation cells (APCs), including macrophages and dendritic cells, secrete IL-10, a crucial regulatory cytokine for regulating host responses to infections. Interleukin-10 (IL10) can control intestinal endocrine activity and is vital in tolerance to self and mucosal antigens [65]. In addition, IL-10 can suppress INF production, a pro-inflammatory cytokine, to prevent further inflammation [66,67]. The current study showed increased serum levels of lysozymes, IL10, and C3 in GLU-supplemented groups. β-glucan can activate macrophages, increase lysozyme activity, and regulate the release of interleukins and tumor necrosis factor-alpha (TNF-α) in macrophages and dendritic cells [68].

Furthermore, β-glucan induces B cells to produce IgG immunoglobulin by binding complement receptor type three (CR3), Dectin-1, or TLRs on neutrophils, macrophages, natural killer cells, and dendritic cells, and finally encourages the body to produce an immune response [69]. Ross and Větvička [70] reported that soluble β-glucan could activate CR3. There is proof that β -glucan can boost humoral immunity by triggering T-cells [71]. Glucans increase the release of lysozyme from broiler phagocytic cells, which enhances non-specific immunity [72].

After antigen recognition, the T-cell co-receptor CD3 (cluster of differentiation 3) starts a signaling cascade that activates both helper and cytotoxic T cells [73]. A B-lymphocyte surface antigen, CD20, regulates B-cell activity, differentiation, and proliferation [74]. In the current study, immunohistochemical examination showed a solid immunostaining response against CD3 and CD20 antibodies in the spleen of birds fed GLU-supplemented diets in a level-dependent manner. The expression of specific cell surface biomarkers allows for the identification of lymphocyte subpopulations. The obtained results in this study proved the immunomodulating effects of 1,3-β-glucans when it was supplemented in the diets of broiler chickens.

## 5. Conclusions

From the obtained results, we can conclude that 1,3-β-glucan is a suitable feed additive in broilers’ diets. The optimal level for 1,3-β-glucan dietary supplementation was 120 mg Kg^−1^. Dietary 1,3-β-glucan could improve the development and integrity of the intestine and enhance the bird’s immune status by boosting both humoral and cellular immunity. Moreover, it has a hypolipidemic impact and improves the hormonal profile of birds for better metabolism and growth, as represented by higher T3, T4, and growth hormones. However, these positive effects did not translate to increased body weights in birds.

## Figures and Tables

**Table 1:** Basal diets chemical composition as a fed basis (%).

Constituents	Starter	Grower	Finisher
Corn gluten meal 60% CP	3.7	5.5	5.89
Corn 7.5% CP	56	59	62.2
Soybean meal 48% CP	33.555	28.175	23.8
Soy oil	2.2	3	4
Sodium bicarbonate	0.25	0.25	0.25
Di-calcium phosphate 18%	1.5	1.4	1.3
Calcium carbonate	1.2	1.2	1.1
Broiler premix *	0.3	0.3	0.3
Salt	0.15	0.15	0.15
L-LYSINE HCL 78%	0.47	0.45	0.4
DL-methionine 98%	0.4	0.3	0.33
L-THREONINE 98.5%	0.1	0.1	0.1
Choline 60 veg	0.07	0.07	0.07
Phytase	0.005	0.005	0.005
Antimycotoxin	0.1	0.1	0.1
Chemical analysis			
Moisture	12	12.10	11.6
ME poultry (kcal/kg)	3001.09	3100.8	3200.22
Crude protein	23.00	21.67	20.05
Methionine	0.72	0.61	0.62
Lysine	1.47	1.31	1.16
Calcium	0.94	0.90	0.83
Av. Phosphorus	0.48	0.44	0.41

* Premix per kg of diet: vitamin E–10 mg; vitamin A–1500 IU; vitamin D3–200 IU; vitamin K3–0.5 mg; thiamine–1.9 mg; riboflavin–3.7 mg; pantothenic acid–10 mg; folic acid–0.55 mg; niacin–35 mg; cobalamin–0.01 mg; pyridoxine–3.5 mg; biotin–0.15 mg; Se–0.15 mg; Fe–80 mg; Mn–60 mg; Zn–40 mg; Cu–8 mg; I–0.35 mg.

**Table 2:** The impact of dietary 1,3-β-glucan on the growth of broiler chickens.

	GLU0	GLU50	GLU100	GLU150	SEM	Linear	Quadratic
IBW (g)	97	97.50	97.71	97.50	0.181	0.09	0.122
Starter period (4–10 days)							
BW (g)	333	329	330	327	1.60	0.342	0.930
BWG (g)	236	231	232	229	1.55	0.486	0.926
FI (g)	259	259	262	264	2.49	0.469	0.866
FCR	1.11	1.12	1.13	1.15	0.0123	0.262	0.803
Grower period (11–23 days)							
BW (g)	1136	1154	1182	1137	12.1	0.794	0.248
BWG (g)	804	825	852	810	11.4	0.673	0.214
FI (g)	1075 ^b^	1185 ^ab^	1217 ^a^	1113 ^ab^	21.1	0.294	0.006
FCR	1.34	1.44	1.43	1.37	0.0198	0.543	0.057
Finisher period (24–35 days)							
BW (g)	1925	1967	2010	1957	25.4	0.594	0.420
BWG (g)	789	813	828	820	14.3	0.473	0.628
FI (g)	1333 ^b^	1600 ^a^	1563 ^ab^	1479 ^ab^	38.9	0.143	0.013
FCR	1.69	1.97	1.89	1.81	0.0416	0.367	0.021
Overall performance							
BW (g)	1925	1967	2010	1957	25.4	0.594	0.420
BWG (g)	1826	1869	1912	1859	25.5	0.583	0.414
FI (g)	2667 ^b^	3044 ^a^	3043 ^a^	2856 ^ab^	58.5	0.158	0.009
FCR	1.46	1.63	1.59	1.54	0.0261	0.324	0.025

^a,b^ Means within the same row with different superscripts are statistically different at *p* < 0.05.

**Table 3:** The effect of dietary supplementation with 1,3-β-glucan on the intestinal morphometric measures of broiler chickens.

	GLU0	GLU50	GLU100	GLU150	SEM	Linear	Quadratic
Duodenum							
VH (µm)	2055	1791	1914	1825	42.9	0.118	0.270
VW (µm)	531 ^a^	162 ^d^	377 ^b^	200 ^c^	38.1	0.000	0.000
CD (µm)	394 ^a^	391 ^a^	241 ^c^	328 ^b^	16	0.000	0.000
M.C.T. (µm)	519 ^a^	252 ^c^	264 ^b^	222 ^d^	33.3	0.000	0.000
VH/CD	5.22 ^b^	4.59 ^b^	7.93 ^a^	5.56 ^b^	0.356	0.009	0.018
GCC	25 ^b^	23.5 ^b^	20 ^c^	33.7 ^a^	1.33	0.000	0.000
jejunum							
VH (µm)	1943 ^b^	2123 ^a^	2164 ^a^	2015 ^ab^	132	0.167	0.001
VW (µm)	443 ^a^	304 ^b^	245 ^b^	303 ^c^	18.9	0.000	0.000
CD (µm)	352 ^b^	267 ^d^	280 ^c^	385 ^a^	12.7	0.000	0.000
M.C.T. (µm)	237 ^c^	254 ^b^	219 ^d^	284 ^a^	6.3	0.000	0.000
VH/CD	5.52 ^b^	7.96 ^a^	7.72 ^a^	5.24 ^b^	0.323	0.048	0.000
GCC	15 ^b^	26 ^a^	27 ^a^	25.3 ^a^	1.26	0.000	0.000
ileum							
VH (µm)	1844 ^c^	1895 ^c^	1990 ^b^	2126 ^a^	29.5	0.000	0.080
VW (µm)	292 ^b^	289 ^b^	311 ^a^	239 ^c^	7.10	0.000	0.000
CD (µm)	317 ^a^	252 ^b^	215 ^d^	222 ^c^	18.9	0.000	0.000
M.C.T. (µm)	286 ^a^	226 ^c^	234 ^c^	267 ^b^	6.44	0.004	0.000
VH/CD	5.81 ^c^	7.52 ^b^	9.25 ^a^	9.59 ^a^	0.392	0.000	0.000
GCC	22 ^b^	21.3 ^b^	16.3 ^c^	23.3 ^a^	0.717	0.557	0.000

^a,b,c,d^ Means within the same row with different superscripts are statistically different at *p* < 0.05.

**Table 4:** The impact of dietary 1,3-β-glucan on serum biochemical parameters of broiler chickens.

	GLU0	GLU50	GLU100	GLU150	SEM	Linear	Quadratic
T3 (ng/mL)	3.43 ^b^	4.23 ^ab^	4.89 ^a^	4.99 ^a^	0.228	0.004	0.274
T4 (ng/mL)	19.4 ^b^	20.9 ^b^	23.3 ^a^	24.6 ^a^	0.641	0.000	0.892
GH (ng/mL)	2.83 ^b^	4.40 ^a^	5.03 ^a^	5.23 ^a^	0.304	0.000	0.029
Glucose (mg/dL)	340	342	345	342	1.52	0.483	0.535
Leptin (ng/mL)	2.18	1.63	1.63	1.97	0.119	0.544	0.085

^a,b^ Means within the same row with different superscripts are statistically different at *p* < 0.05.

**Table 5:** The impact of dietary 1,3-β-glucan on the lipid profile of broiler chickens.

	GLU0	GLU50	GLU100	GLU150	SEM	Linear	Quadratic
TG (mmol/L)	1.27 ± 0.003 ^a^	1.16 ± 0.03 ^b^	1.16 ± 0.02 ^b^	1.16± 0.03 ^b^	0.0167	0.011	0.048
TC (mmol/L)	3.55 ± 0.01 ^a^	3.34 ± 0.03 ^b^	3.40 ± 0.07 ^ab^	3.41 ± 0.03 ^ab^	0.0288	0.087	0.024
HDL-C (mmol/L)	1.98 ± 0.01	2.07 ± 0.07	2.11 ± 0.03	2.14 ± 0.02	0.0250	0.019	0.452
LDL-C (mmol/L)	1.33 ± 0.02 ^a^	1.05 ± 0.08 ^b^	1.06 ± 0.07 ^b^	1.04 ± 0.03 ^b^	0.0440	0.010	0.045
VLDL-C (mmol/L)	0.24 ± 0.01	0.22 ± 0.01	0.23 ± 0.01	0.23 ± 0.01	0.0041	0.564	0.364

^a,b^ Means within the same row with different superscripts are statistically different at *p* < 0.05.

**Table 6:** The impact of dietary 1,3-β-glucan on the immune status of broiler chickens.

	GLU0	GLU50	GLU100	GLU150	SEM	Linear	Quadratic
Lysozyme (µg/mL)	137 ^b^	184 ^a^	182 ^a^	190 ^a^	6.65	0.000	0.001
IL10 (pg/mL)	1.70 ^b^	3.27 ^a^	4 ^a^	4.20 ^a^	0.312	0.000	0.018
C3 (mg/dL)	1.09 ^c^	1.15 ^b^	1.23 ^a^	1.24 ^a^	0.0191	0.000	0.103
TP (g/dL)	3.68 ^b^	4.49 ^ab^	4.69 ^a^	5.13 ^a^	0.178	0.001	0.353
ALB (g/dL)	1.29	1.36	1.92	1.74	0.130	0.118	0.631
Globulin (g/dL)	2.39	3.13	2.77	3.39	0.187	0.131	0.857

^a,b,c^ Means within the same row with different superscripts are statistically different at *p* < 0.05.

## Data Availability

The datasets used and analyzed during the current study are available from the corresponding author on reasonable request.
